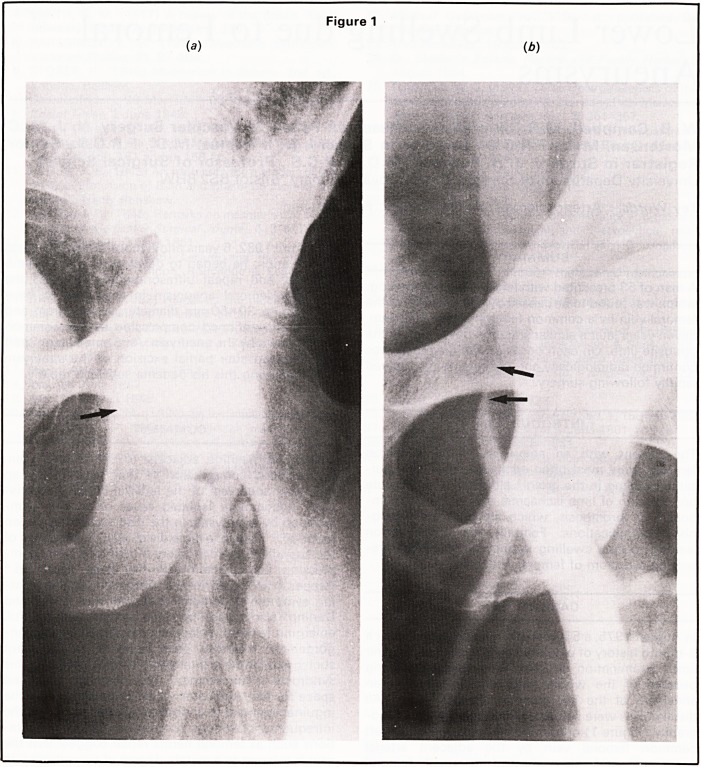# Lower Limb Swelling Due to Femoral Aneurysms

**Published:** 1983-04

**Authors:** W. B. Campbell, N. J. McC Mortensen, N. I. Ramus, J. H. Peacock

**Affiliations:** University Department of Surgery, Bristol Royal Infirmary, Bristol BS2 8HW; University Department of Surgery, Bristol Royal Infirmary, Bristol BS2 8HW; University Department of Surgery, Bristol Royal Infirmary, Bristol BS2 8HW; University Department of Surgery, Bristol Royal Infirmary, Bristol BS2 8HW

**Keywords:** Arteriosclerosis, Femoral artery, Femoral vein

## Abstract

A man of 53 presented with left lower limb swelling, which was found to be caused by compression of the femoral vein by a common femoral artery aneurysm. Seven years later a similar situation developed in the opposite limb. On each occasion the diagnosis was confirmed radiologically and the symptoms resolved rapidly following surgery.


### Lower Limb Swelling due to Femoral Aneurysms

**W. B. Campbell, M.R.C.P., F.R.C.S., Research Fellow in Vascular Surgery, N. J. McC Mortensen, M.D., F.R.C.S., Lecturer in Surgery, N. I. Ramus, M.D., F.R.C.S., Senior Registrar in Surgery, J. H. Peacock, M.D., F.R.C.S., Professor of Surgical Science**
University Department of Surgery, Bristol Royal Infirmary, Bristol BS2 8HW

*Key Words*: Arteriosclerosis; Femoral artery; Femoral vein

### SUMMARY

A man of 53 presented with left lower limb swelling, which was found to be caused by compression of the femoral vein by a common femoral artery aneurysm. Seven years later a similar situation developed in the opposite limb. On each occasion the diagnosis was confirmed radiologically and the symptoms resolved rapidly following surgery.

### INTRODUCTION

The patient with an aneurysm of the common femoral artery most often complains of a local pulsatile swelling in the groin,[1] although a more acute presentation of limb ischaemia may result from embolism or thrombosis, which are both well recognised complications. Femoral vein compression leading to limb swelling is uncommon as the presenting symptom of femoral aneurysm.

### CASE REPORT

In August 1975, a 53-year-old man presented with a 3-month history of painless swelling of the left lower limb. Examination at that time revealed pitting oedema of the whole limb, and in addition an aneurysm of the left common femoral artery. All distal pulses were palpable. Arteriography and venography (Figure 1) confirmed compression of the left common femoral vein by the adjacent arterial aneurysm, and also showed a small femoral aneurysm on the opposite side. The left femoral aneurysm was excised, and continuity restored by an 8 mm woven Dacron graft. He subsequently attended for regular outpatient review and was still asymptomatic in October 1979, when an ultrasound scan showed his right femoral aneurysm to measure 20×25 mm in diameter (anteroposterior×transverse).

Correspondence to W. B. Campbell.

In April 1982, 6 years and 8 months after his initial presentation, he began to develop right lower limb oedema, and repeat ultrasonography showed the common femoral aneurysm in that limb to have enlarged to 30×50 mm diameter. A venogram and C.T. scan confirmed compression of the common femoral vein by the aneurysm. An 8 mm Dacron graft was inserted after partial excision of the aneurysm sac. Following this his oedema subsided rapidly.

### COMMENT

Despite its position adjacent to the femoral vein, symptomatic compression of that structure by an enlarging aneurysm of the femoral artery is surprisingly uncommon. Isolated cases of venous compression by aneurysms in the iliac region have been reported,[2, 3] and at a more distal level an aneurysm of the superficial femoral artery in Hunter's canal compressing the adjacent vein has been described.[4] Pappas *et al.*[5] listed venous congestion as a presenting symptom of femoral aneurysm and Cutler and Darling[6] also stated that some patients with painful, enlarging femoral aneurysms developed venous engorgement. However, other series fail to document such cases, and it is interesting to speculate why this syndrome is uncommon. The relatively unlimited space for free expansion of an aneurysm below the inguinal ligament is undoubtedly one factor, but the infrequency of venous congestion following operations such as femoral hernia repair suggest that the vein is tolerant to a certain degree of compression in the restricted space behind the inguinal ligament. Despite severe compression in this case, no distal thrombosis had occurred, although this was suspected clinically at the time of his second presentation. Early recourse to venography avoided incorrect treatment with anticoagulants.

It is important to make a distinction between true aneurysms of the common femoral arteries, and false aneurysms which have become more common as a result of aortofemoral grafting procedures for lower

Figure 1

(*a*) (*b*)

limb ischaemia. True aneurysms of the peripheral arteries are quite frequently multiple[1, 5] and those of the femoral artery have been reported as occurring bilaterally in about one-third of cases. Nevertheless, the syndrome of lower limb oedema due to such lesions, first on one side and then the other has not, so far as we are aware, been reported previously.

Femoral vein compression by a common femoral artery aneurysm is an uncommon cause of lower limb swelling. However, once suspected on clinical grounds the diagnosis is readily confirmed radiologically, and response to operative treatment is rapid and permanent.

### REFERENCES

1. CRAWFORD, E. S., DeBAKEY, M. E. and COOLEY, D. A. (1959) Surgical considerations of peripheral aneurysms. *Arch.Surg.* **78**, 236.
2. MARKLOWITZ, A. M. and NORMAN J. C. (1961) Aneurysms of the iliac artery. *Ann.Surg.* **154**, 777.
3. BERNARD, R. W., IMPARATO, A. M. and MUND, A. (1970) Iliac artery aneurysm presenting as acute ilio-femoral vein occlusion. *Vascular Surgery* **4**, 186.
4. BATES, J. D. (1977) Unilateral swelling of a lower extremity secondary to primary arterial disease. *J.Am.Osteop.Ass.* **76**, 688.
5. PAPPAS, G., JANES, J. M., BERNATZ, P. E. and SCHIRGER A. (1964) Femoral aneurysms: a review of surgical management. *JAMA* **190**, 489.
6. CUTLER, B. S. and DARLING, R. C. (1973) Surgical management of arteriosclerotic femoral aneurysms. *Surgery* **74**, 764–773.

## Figures and Tables

**Figure 1 f1:**